# Adsorption and protective behavior of BTAH on the initial atmospheric corrosion process of copper under thin film of chloride solutions

**DOI:** 10.1038/s41598-018-23927-w

**Published:** 2018-04-04

**Authors:** Chenxi Yi, Benfeng Zhu, Yu Chen, Xiaoqing Du, Yumeng Yang, Jiao Liu, Zhao Zhang

**Affiliations:** 1 Department of Chemistry, Zhejiang University, Hangzhou, Zhejiang, 310027 China; 2 Department of Chemical Engineering and Safety, Binzhou University, Binzhou, Shandong 256600 China; 3 College of Chemistry and Chemical Engineering, Hunan University, Changsha, 410082 China

## Abstract

The initial corrosion process of copper and the corrosion resistance mechanism of Benzotriazole under chloride-containing thin electrolyte layer (TEL) was investigated. After theoretical calculation and experimental characterization, the forming process of [Cu(I)BTA]_n_ film was chemically adsorbed on copper surface by Cu-N bond tightly; corrosion rate increased as TEL thickness decreased. Whilst, energy distribution plot of electrochemical noise provided the validity of corrosion type, and the purported corrosion energy (*E*_c_) deduced from electrochemical noise was approximately proportion to corrosion rate (1/*R*_ct_) with and without the anticorrosion film, which denoted the feasibility to determine corrosion rate by nondestructive on-line monitoring electrochemical noise progress.

## Introduction

As architectural materials, copper and its alloys have been widely used in marine industry due to its excellent performance^1,2^. However, it still suffers from serious damage during its long time serving processes, especially in marine environments^3,4^.

The predominant ion in seawater is Cl^−^, which is well known for stimulation of metal corrosion. The corrosion mechanism of copper influenced by chloride have been discussed by many researchers^3,5^. According to the critical review by Kear and his coworkers^6^, the anodic reaction mechanisms during copper corrosion in the presence of chloride ions is^7–9^:

1 $${\rm{Cu}}+{{\rm{Cl}}}^{-}\mathop{\leftarrow }\limits_{{k}_{-1}}\mathop{\to }\limits^{{k}_{1}}{\rm{CuCl}}+{e}^{-}$$

while, the cathodic reaction is as follows^10^:

2 $$\frac{1}{4}{{\rm{O}}}_{2}+\frac{1}{2}{{\rm{H}}}_{2}{\rm{O}}+{e}^{-}\mathop{\to }\limits^{{k}_{2}}{{\rm{OH}}}^{-}$$

For the initial corrosion process of copper, the main corrosion product is Cu_2_O^11,12^ via:

3 $${\rm{CuCl}}+{{\rm{OH}}}^{-}\mathop{\to }\limits^{{k}_{3}}\frac{1}{2}{{\rm{Cu}}}_{2}{\rm{O}}+\frac{1}{2}{{\rm{H}}}_{2}{\rm{O}}+{{\rm{Cl}}}^{-}$$

Benzotriazole (BTAH), one of the most efficient inhibitors for copper, is the most highly stressed subject of numerous scientific studies^13–16^, and its possible inhibition mechanism has also been proposed: the formation of an adsorption layer of BTAH^17,18^ or a complex polymeric film of [Cu(I)BTA]_n_^19–21^ on copper surface via reaction^22^,

4 $${\rm{n}}{({\rm{BTAH}})}_{{\rm{ads}}}+{\rm{nCuCl}}\to {[{\rm{Cu}}({\rm{I}}){\rm{BTA}}]}_{{\rm{n}}}+{{\rm{nH}}}^{+}+{{\rm{nCl}}}^{-}$$

Metal corrosion behavior in atmospheric environments is significantly different from that in bulk solutions^10,23–25^. Atmospheric corrosion is an electrochemical process occurring on a metal surface covered with a thin electrolyte layer (TEL). TEL thickness markedly affects the corrosion-related processes, such as the mass transport of the dissolved oxygen and the accumulation of corrosion products^26,27^. Yi *et al*.^27^ studied the atmospheric corrosion behavior of PCB-ENIG under the adsorbed thin electrolyte layer and found that the cathodic current density in the solution was greater than that under TEL, and decreased with the thinning of TEL film. Moreover, the controlling step of the oxygen reduction process transferred from the cathodic to the anodic process in the extremely thin liquid film.

Nowadays, a wide variety of electrochemical measurements coupled with surface analytical^28^ and spectroscopic techniques^29^ have been adopted to study the metal corrosion processes. However, it is hard to detect the corrosion *in-situ* and non-destructive simultaneously. Corrosion processes are associated with electrochemical metal dissolution, involving charge transfer that generate spontaneous fluctuations in current and potential. These fluctuations are defined as electrochemical noise (EN), whose measurements have received considerable attentions^30–32^. EN has been regarded as a powerful electrochemical technique and has been successfully utilized to investigate the corrosion process^33–35^ and electrodeposition process^36^. The prime attraction of EN technique in corrosion study is its *in-situ* monitoring the early initiation corrosion process with high sensitivity and no damage, whereas the traditional techniques (such as the polarization tests and the electrochemical impedance spectroscopy, etc.) often cause unexpected damage by introducing the external perturbation into the investigated electrochemical system, and may provide the corrosion information with some possible artifacts.

The frequency contribution of each EN individual transient leaves a specific signature, or “fingerprint” that can provide information on the nature of the related corrosion process^36–39^. Fast Wavelet Transformation technique, as a kind of discrete wavelet transform, describes the EN curves at several time-scale in so-called crystals, and the relative energy contribution from each crystal can be visualized in an energy distribution plot (EDP)^36^. By using the so-called RP-EDP (the replotted energy distribution plot), which discounts the contribution of smooth coefficient set from the overall ensemble signal energy, some researches took an insight into the relationship between the position of the maximum relative energy and the dominant process in certain corrosion events^40^, and found that EDP can provide the useful information about the transformation of the typic corrosion type of Al in NaCl solutions^41^ and AISI 1020 steel corrosion in seawater^42^ during their corrosion processes. However, the quantitative or even the semiquantitative information about the corrosion process (especially the corrosion severity) from the viewpoint of noise energy has not been reported.

The aim of this paper is to investigate the inhibition behavior of well-known BTAH on Cu mainly using electrochemical impedance spectroscopy (EIS) and EN techniques, especially to quantitatively probe into the relationship between the corrosion severity of Cu and its corresponding corroding noise energy. Meanwhile, the corrosion mechanism of Cu in the investigated corroding conditions was verified from the EIS theoretical calculations. These yields should increase the discrimination ability between the corrosion sensitivity and the electrochemical noise energy.

## Methods

### Materials

The corrosion process of pure copper in 3.5 wt.% NaCl electrolyte was studied. The working electrode was mechanically cut and embedded into Teflon, leaving an exposed area of 0.5 cm^2^ as working surface. Prior to each experiment, the samples were abraded gradually using sand paper from 400 to 1200 grit, polished with 2.5 μm diamond paste. Subsequently, the surface was rinsed with distilled water by ultrasonic cleaner about 3 min (KQ5200B, Youyi instrument Co., Ltd., China), degreased with acetone, and finally dried in a cool N_2_ flow.

[Cu(I)BTA]_n_ film on Cu was fabricated as follows^15,16^: the copper electrode was pre-immersed into the solution containing 0.1 mM BTAH and 4.0 M NaCl at 60 °C for 3 hours, then ultrasonic cleaned using distilled water twice to eliminate the obstruction caused by the absorption of BTAH molecule layer, and finally drying in cool N_2_ flow again. In this way, the copper substrate would be covered by a thin layer of [Cu(I)BTA]_n_ film. Hereinafter, the copper electrode covered with [Cu(I)BTA]_n_ film is designated as CuBTA, while those without BTAH pretreatment is simply named as Cu.

After that, CuBTA and Cu were fixed under a layer of thin electrolyte containing 3.5 wt.% NaCl, and all the measurements were performed at the temperature of 20 ± 1 °C.

### Thin electrolyte layer set-up

**Figure 1:**
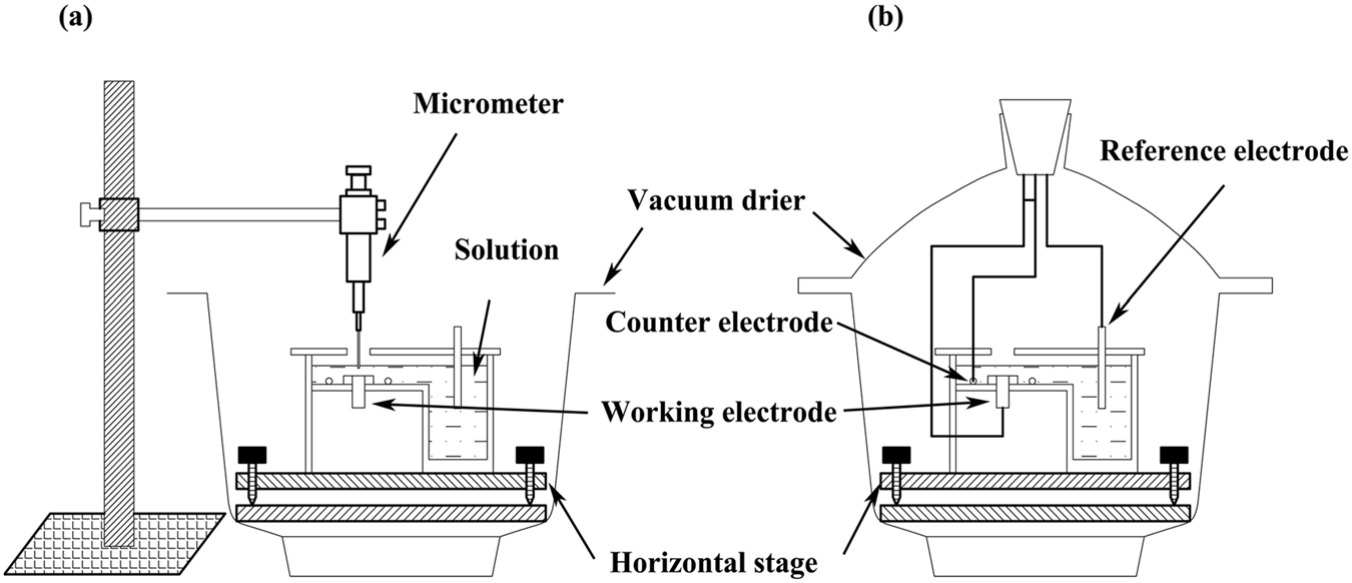
Schematic diagram for (**a**) determination of TEL thickness and (**b**) electrochemical measurement in corrosion study. The electrochemical cell, which was placed on a horizontal stage in constant humidity chamber, was adjusted to the horizontal level using a water level.

The schematic diagram of TEL is president in Fig. 1. The working electrode was firmly installed in the cell, leaving only the upper surface exposed. A platinum wire (0.5 mm diameter) was fixed around the working electrode and positioned below the exposed surface and served as the counter electrode. A saturated calomel electrode (SCE) connected with salt bridge was inserted into the bulk solution and used as the reference electrode. The electrochemical cell, which was placed on a horizontal stage in constant humidity chamber, was adjusted to the horizontal level using a water level.

The TEL thickness was determined by equipment consisting of a sharp Pt needle and an iron support with a micrometer, as shown in Fig. 1a. The TEL thickness was determined by equipment consisting of a sharp Pt needle and an iron support with a micrometer. The first value on the micrometer was recorded at once when the Pt needle touched the electrode surface. After infusing NaCl solution into the cell, the second value was recorded when the Pt needle touched the electrolyte surface.

A constant humidity chamber with an electrochemical cell was completely covered with a lid (Fig. 1b) and a NaCl solution of the same concentration as the test solution was placed at the bottom of the chamber to maintain the stability of the TEL thickness for long immersion times during the electrochemical measurements.

### Electrochemical measurements

Electrochemical measurements were conducted on an electrochemical workstation (CHI630 CH Instruments, Inc.) and all potentials were reported with respect to SCE. Tafel curve tests under various TEL thickness were conducted with a sweep rate of 1 mV/s. For the linear polarization measurements, a sweep range of −5 to +5 mV vs. OCP (open circuit potential) at a sweep rate of 1 mV/s was used, and the polarization resistance (*R*_p_) was determined from the slope of *E* vs. *i* curve in the vicinity region of the corrosion potential. EIS measurements were conducted in the 100 kHz to 10 mHz frequency range at the OCP with ±5 mV potential perturbation. The component values of EIS equivalent circuit were calculated using Z-view 3.1 software.

EN was *in-situ* recorded using GP Amp analyzer (A D Instruments Pty Ltd., Australia). The interval sample time was 0.25 second, by which most usual corrosion processes can be detected. The frequency window of the observation can be calculated roughly by^43^:

5 $$({C}_{1}^{l},{C}_{2}^{l})=({2}^{l}{\rm{\Delta }}t,{2}^{l-1}{\rm{\Delta }}t)$$

Where *l* is the number of the crystal, and ∆t is the sampling interval of 0.25 s. During the EN measurements, the experimental device was shielded in a Faradaic cage. The EN measurements were performed in a quiescent solution at 20 ± 1 °C without stirring, which was also controlled by a thermostatically water bath. The Spectra were graphed using Origin 8.0 (OriginLab, Northampton, MA), and the energy values were calculated by Matlab R2014b software (The MathWorks, Inc.).

### XPS imaging

X-ray photoelectron spectroscopy (XPS) analyses were performed on a VG ESCALAB MARK II spectrometer with the Mg Kα radiation (1253.6 eV), operating at constant pass energy mode at 50 eV. The surface charging effect was corrected by fixing the C 1s peak at a binding energy of 284.6 eV. The constitution of each peak was processed using XPSPEAK Version 4.0 software.

### FTIR spectroscopy

FTIR were recorded with a Nexus 670 (Nicolet) FT-IR spectrometer. 32 scans were performed for each spectrum with a resolution of 2 cm^−1^.

### SEM

SEM (EDS) were performed by a SU-8010 Scanning Electron Microscope (Hitachi) coupled with an Oxford X-Max^N^ 50mm^2^ detector, using the low vacuum mode and the accelerating voltage of 15.0 kV.

### Data availability

The datasets generated during the current study are available from the corresponding author on reasonable request.

## Results and Discussion

### Characterization of BTAH adsorption film

**Figure 2:**
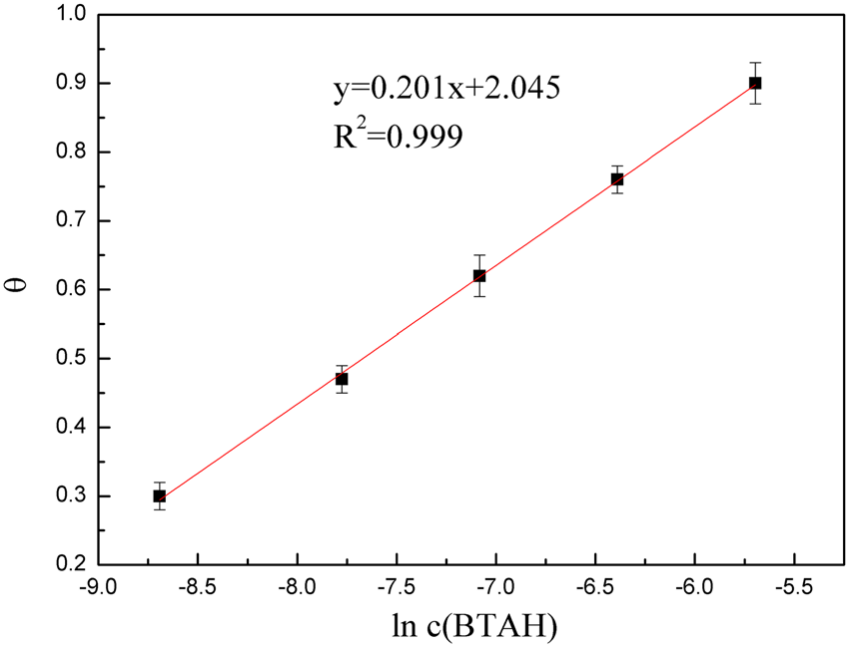
Temkin adsorption isotherm plots for BTAH adsorption on Cu in the solution containing 4.0 M NaCl at 60 °C. The fitting line is listed and R^2^ equals to 0.999.

The surface coverage degree (*θ*_3_) of BTAH on copper could be obtained by weight loss measurement:

6 $${\theta }_{3}=\frac{{\rm{\Delta }}{m}_{0}-{\rm{\Delta }}{m}_{1}}{{\rm{\Delta }}{m}_{0}}$$

where Δ*m*_0_ and Δ*m*_1_ are the mass loss in the absence and presence of the BTAH. Figure 2 shows the relationship between BTAH concentration and surface coverage. For BTAH, the plot of θ_3_ against logarithm of inhibitor concentration gives straight line, which is the characteristics of the Temkin adsorption isotherm given by^44,45^:

7 $$\exp (-2{\rm{\alpha }}\theta )={{\rm{K}}}_{0}{\rm{c}}$$

where α is the molecular interaction parameter, θ is the surface coverage degree, c is the inhibitor concentration in the solution, K_0_ is the equilibrium constant of the adsorption process which is related to the standard free energy ${\rm{\Delta }}{G}_{ads}^{0}$ of adsorption,

8 $${\rm{K}}=\frac{1}{55.5}\exp (-\frac{{\rm{\Delta }}{G}_{ads}^{0}}{RT})$$

Generally^46^, ${\rm{\Delta }}{G}_{ads}^{0}$ values of up to −20 KJ·mol^−1^ are consistent with the physical adsorption, while those more negative than −40 KJ·mol^−1^ involve charge sharing or transfer from inhibitor molecules to metal, i.e. chemisorption. The calculated ${\rm{\Delta }}{G}_{ads}^{0}$ equals to −40.64 KJ·mol^−1^ at 60 °C, therefore the BTAH is chemisorbed onto copper surface.

**Figure 3:**
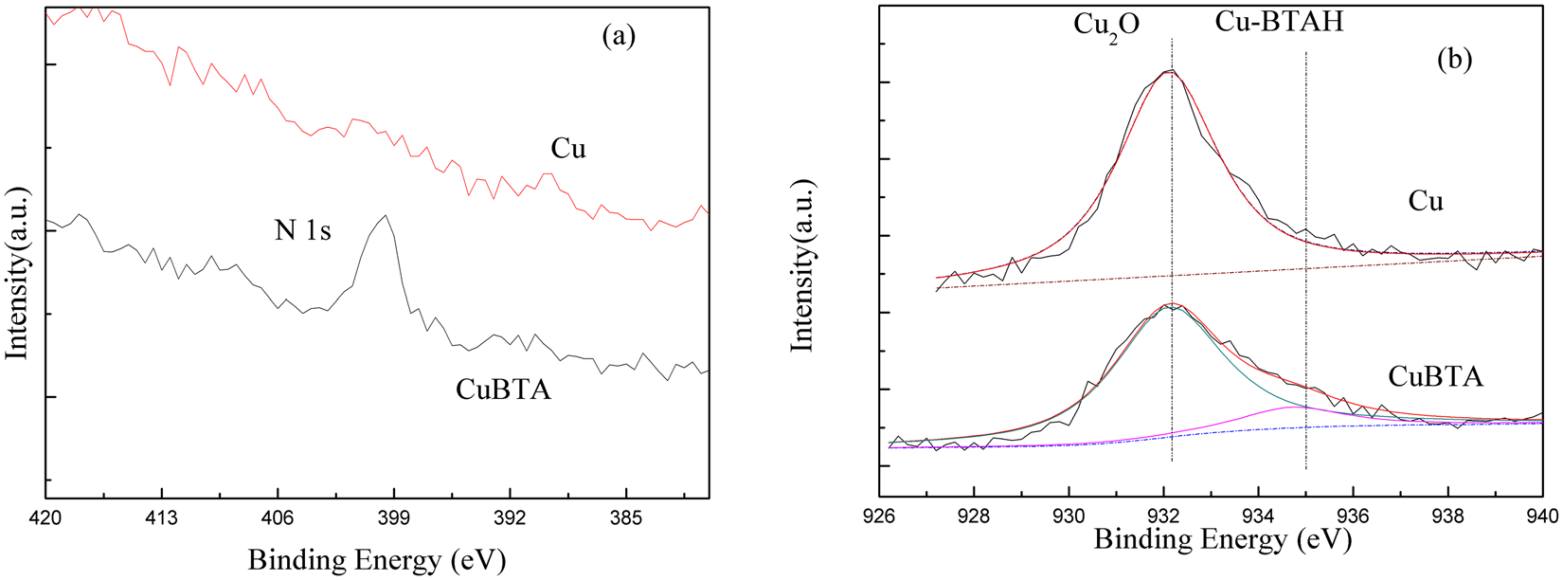
The XPS spectra of CuBTA and Cu after 100 min’s corrosion in 3.5 wt.% NaCl: (**a**) N 1 s and (**b**) Cu 2p_3/2_. The N1s peak at a binding energy of 399.6 eV and the Cu2p peak at 932.2 eV, and the surface charging effect was corrected by fixing the C 1s peak at a binding energy of 284.6 eV.

Figure 3 shows the XPS spectra of CuBTA and Cu surface. The presence of [Cu(I)BTA]_n_ film is confirmed by the N1s peak at a binding energy of 399.6 eV (Fig. 3a), which matches with nitrogen bound to phenyl groups or conjugated nitrogen^47^. The Cu2p peak at 932.2 eV (Fig. 3b) is attributed to the Cu and Cu_2_O that is hardly distinguished by 0.1 eV binding energy shift^48^. Based on the binding energies of Cu2p_3/2_ peak components^49^, no CuO is detected when Cu is not pretreated with BTAH. Whereas, on CuBTA, a second component is detected on the Cu2p_3/2_ peak spectra at a binding energy of 934.7 eV, which originates from [Cu(I)BTA]_n_ film^50^.

**Figure 4:**
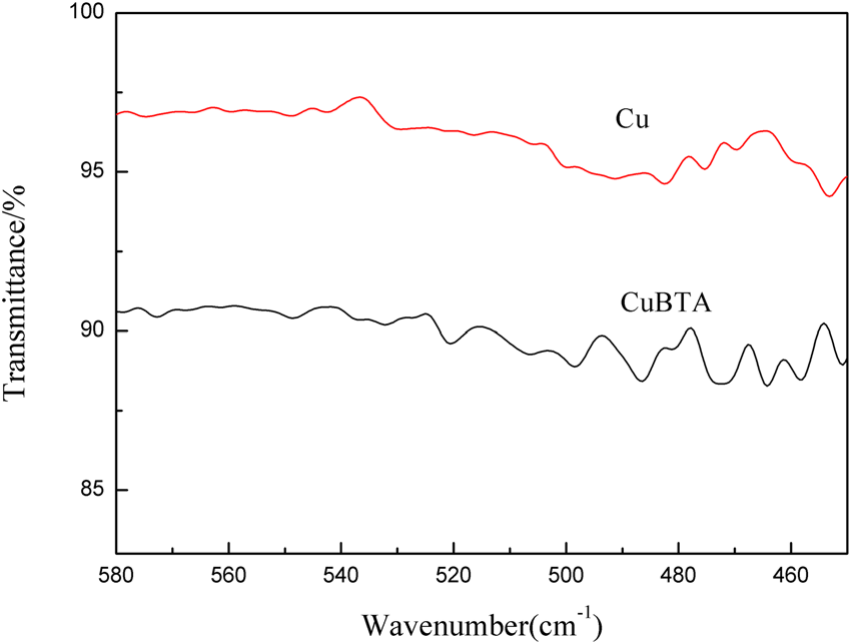
FTIR spectra of CuBTA and Cu after 100 min’s corrosion under 100 µm TEL of 3.5 wt.% NaCl. The peak located in 520 cm^−1^ has been attributed to the stretching vibration of Cu-N bond.

The formation of Cu-N bond can also be confirmed by the FTIR analysis of copper surface on CuBTA after 100 min’s corrosion (Fig. 4). Generally, the peaks located in 520–570 cm^−1^ have been attributed to the stretching vibration of Cu-N bond^51,52^, therefore, the obvious peak emerged at 520 cm^−1^ on CuBTA indicates that BTAH has been successfully chemisorbed onto Cu substrate via the formation of Cu-N atom bond.

### Corrosion behavior under TELs

**Figure 5:**
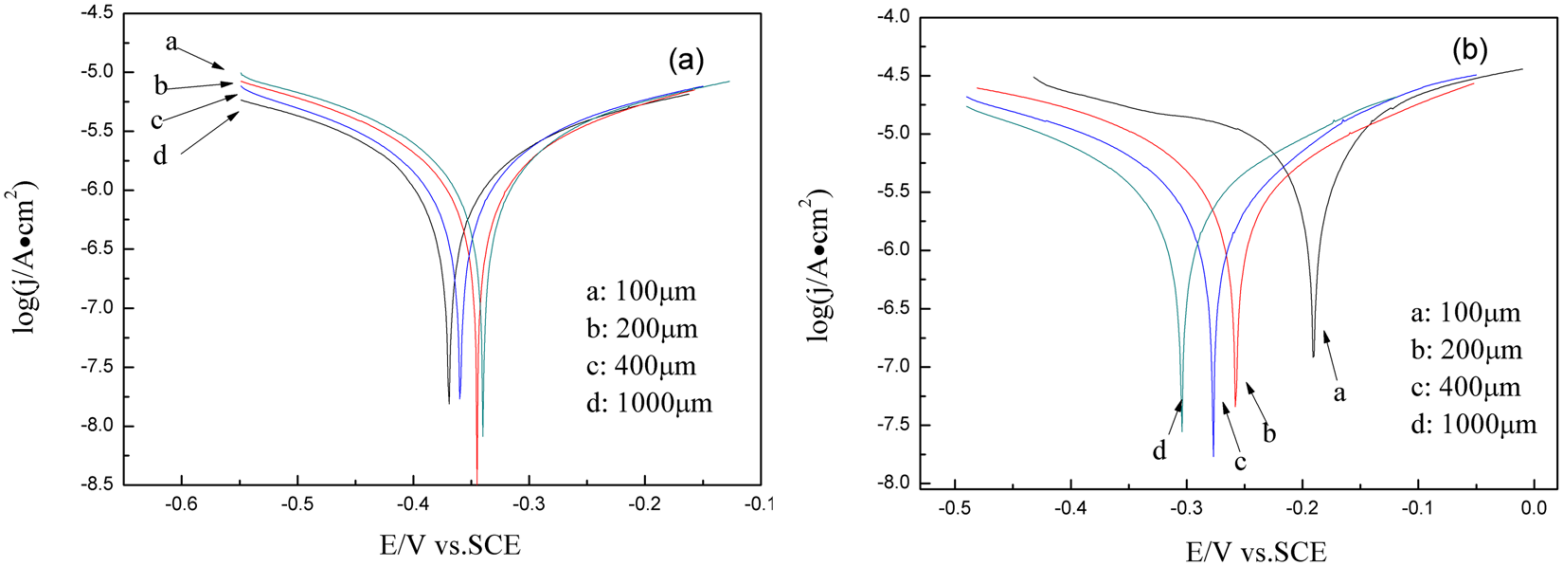
Tafel polarization curves of (**a**) Cu and (**b**) CuBTA under various thickness of TELs of 3.5 wt.% NaCl at a scan rate of 1 mV/s.

Figure 5 shows the Tafel curves of CuBTA and Cu under various thickness of TEL, respectively. According to Stern-Geary equation^53^, the corrosion current (*I*_corr_) that is also simply represented as the corrosion rate can be calculated,

9 $${I}_{{\rm{corr}}}=\frac{B}{{R}_{{\rm{p}}}}=\frac{{b}_{a}{b}_{c}}{2.3({b}_{a}+{b}_{c})}\cdot \frac{1}{{R}_{{\rm{p}}}}$$

where *R*_p_ is the so-called polarization resistance, *b*_a_ and *b*_c_ are the anodic and cathodic Tafel slopes, respectively. Therefore, *R*_p_, *I*_corr_ and other parameters are listed in Table 1.

**Table 1:** Corrosion current densities and Tafel slopes at different thickness electrolyte.

	Thickness/μm	*R*_p_/Ω·cm^2^	*b*_a_/mV·dec^−1^	*b*_c_/mV·dec^−1^	*I*_corr_/μA·cm^−2^
Cu	100	1646	222.5	−353.7	36.08
	200	1875	215.1	−221.5	25.30
	400	2616.5	177.0	−196.1	15.46
	1000	2839	183.2	−195.5	14.48
CuBTA	100	5984	211.3	−214.2	7.530
	200	6153	214.5	−198.0	7.376
	400	6405	205.4	−202.6	7.124
	1000	6731	214.0	−200.0	7.078

The result that *b*_a_ < *b*_c_ for the Cu (Table 1) implies the anodic branch is steeper than the cathodic branch, which indicates that the cathodic process plays more important role in copper corrosion reactions^54^, and also supports the reported viewpoint that the metal atmospheric corrosion process is controlled by cathodic process when the TEL thickness falls in the range of 1 μm ~1 mm^55,56^. However, due to the protective effect of BTAH, *b*_a_ > *b*_c_ for CuBTA except the singular point when TEL thickness is 100 μm (Table 1). Whilst, both the corrosion currents of Cu and CuBTA increase with the decrease of TEL thickness (Table 1), which should be attributed to the higher transfer rate of oxygen across the thinner TEL film and its resulted acceleration on the cathodic reaction^56^.

**Figure 6:**
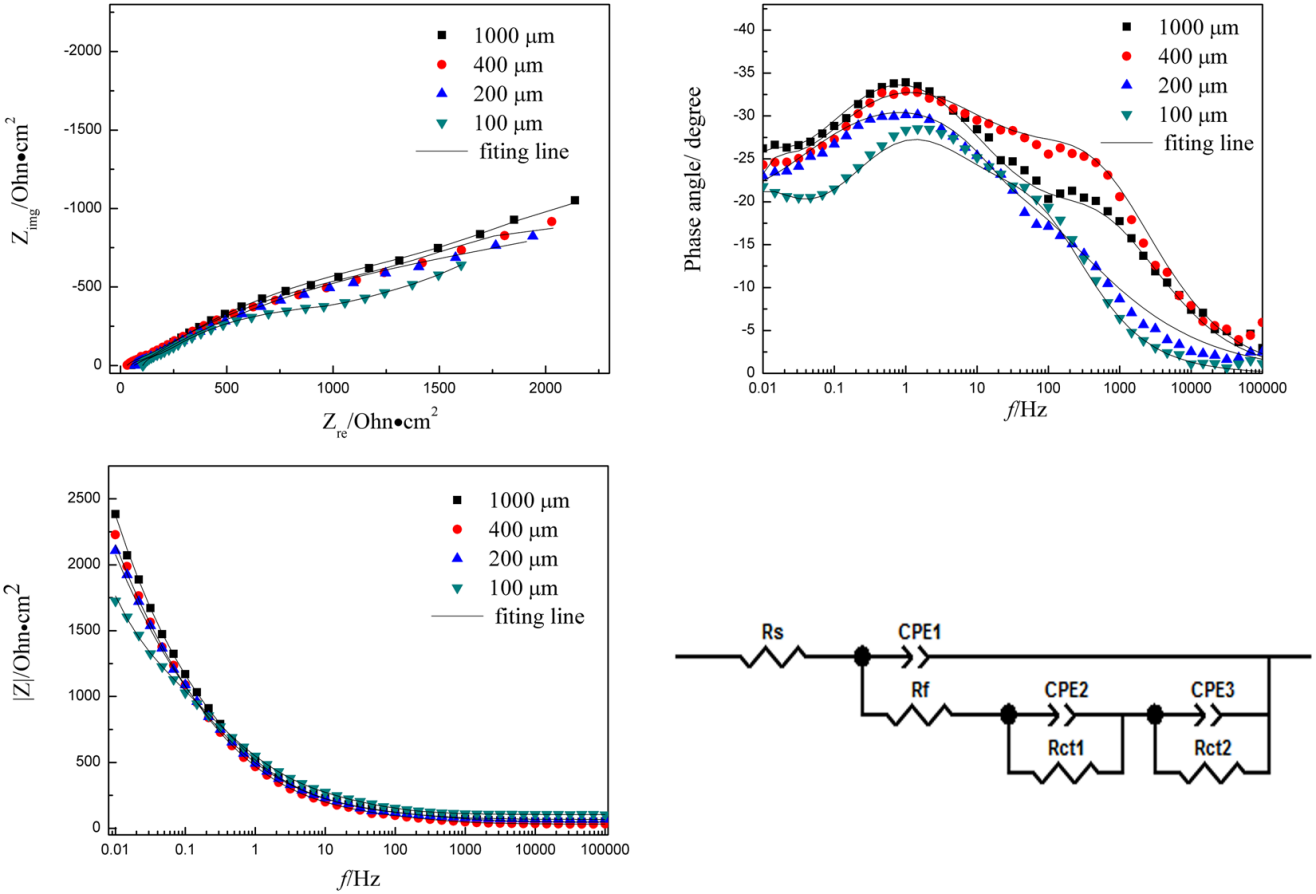
EIS plots of Cu under different thickness of TELs and corresponding equivalent circuit model. Symbols are experimental data and lines are simulated data using the equivalent circuit.

**Figure 7:**
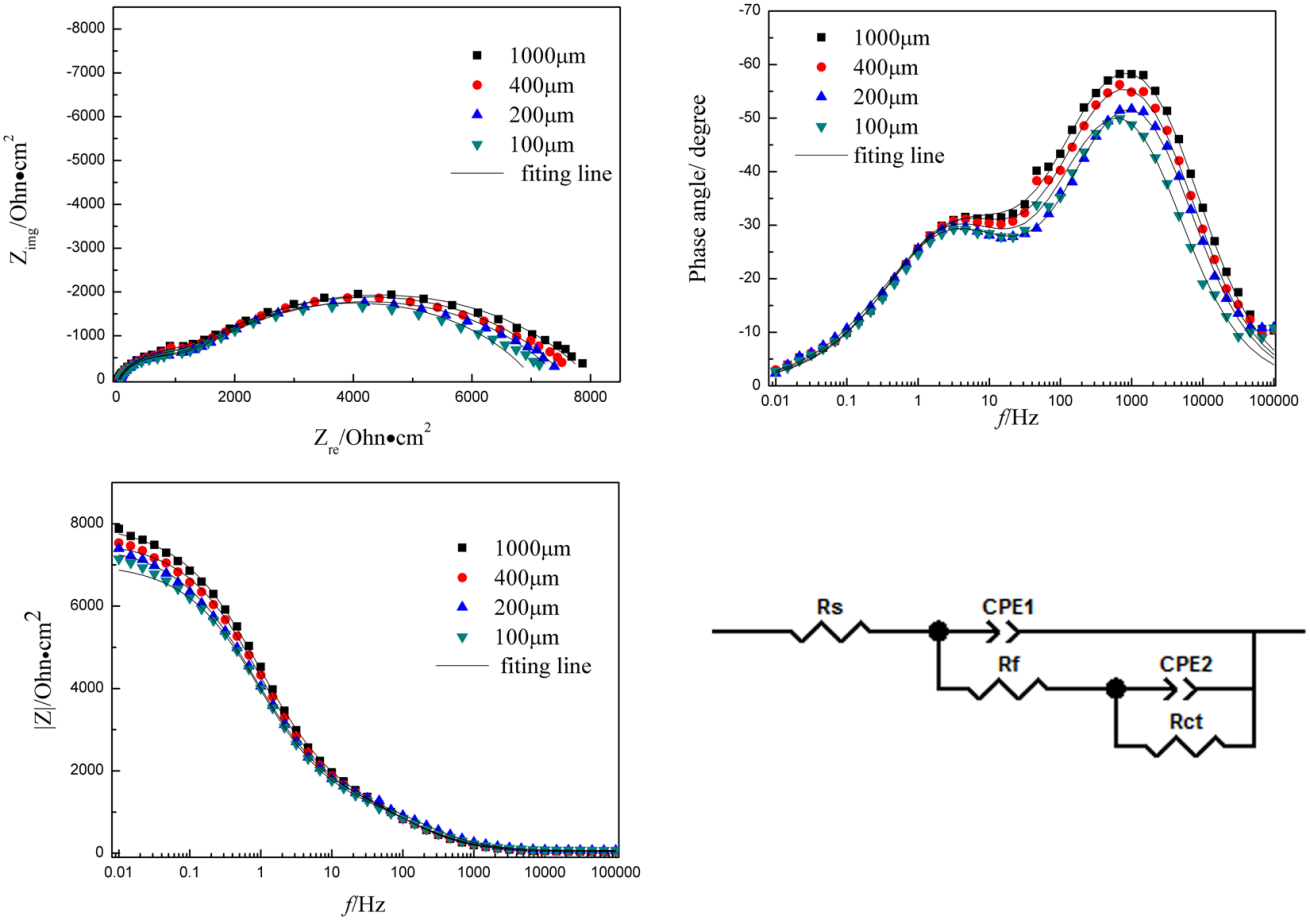
EIS plots of CuBTA under different thickness of TELs and corresponding equivalent circuit model. Symbols are experimental data and lines are simulated data using the equivalent circuit.

Figures 6 and 7 show the EIS plots of Cu and CuBTA under TEL at different thickness, respectively.

The proposal models should be based on the reaction mechanism for analyzation. The proposed reactions equations (1) and (2) steps are electrochemical process, while equation (3) is a chemical process. The rate constants of the elementary steps (*k*_1,_ *k*_−1_ and *k*_2_) obey the Tafel laws:

10 $${k}_{i}={A}_{i}\exp ({b}_{i}E)(i=\pm 1,2)$$

11 $${b}_{i}=nF/2RT$$

where *A*_i_ is the potential-independent pre-exponential rate constant, *E* is overpotential and *n* is the number of electrons involved in the reaction, *k*_i_ is the rate constant. While the rate constant *k* of step 3 is independent of potential.

Here present theoretical considerations for the elucidation of physical models for EIS of the corrosion process. When small ac perturbation signals are applied to a system, the Faradaic current, *I*_F_, the adatom coverage, *θ*_i,_

12 $${\rm{\Delta }}{I}_{F}={(\frac{\partial {I}_{F}}{\partial E})}_{ss}{\rm{\Delta }}E+\sum _{i=1}^{n}{(\frac{\partial {I}_{F}}{\partial {\theta }_{i}})}_{ss}{\rm{\Delta }}{\theta }_{i}$$

The subscript “ss” represent steady state. In which, ${\rm{\Delta }}E=E-{E}_{ss}$, ${\rm{\Delta }}{\theta }_{i}={\theta }_{i}-{({\theta }_{i})}_{ss}$, *i* = 1, 2, …*n*. The Δ corresponds to parameters which oscillate during ac probing.

The whole impedance of the electrode is based on these reactions, the impedance of the whole system:

13 $$Z={R}_{s}+\frac{1}{{Y}_{F}+j\omega {C}_{dl}}$$

14 $${Y}_{F}={\rm{\Delta }}{I}_{F}/{\rm{\Delta }}E$$

in which, *R*_s_ is solution resistance, Cdl is double-layer capacitance (in equivalent circuit model map, we use CPE1 to stand for it), and *Y*_F_ is Faraday admittance^57^, combined with equations (12) and (14), the expression should be:

15 $${Y}_{F}=1/{R}_{f}+\sum _{k=1}^{n}\,{B}_{k}/({a}_{k}+j\omega )$$

In which, *l* stands for the state variable except *E*,

16 $${a}_{i,l}=-{(\frac{\partial {{\epsilon }}_{i}}{\partial {\theta }_{l}})}_{ss}i,l=1,2,\ldots ,n$$

17 $${B}_{l}=\sum _{i=1}^{n}{m}_{i,l}\cdot {b}_{l}\,i,l=1,2,\ldots ,n$$

18 $${m}_{i,l}={(\frac{\partial {I}_{Fi}}{\partial {\theta }_{l}})}_{ss}i,l=1,2,\,\ldots ,n$$

19 $${b}_{l}={(\frac{\partial {{\epsilon }}_{l}}{\partial E})}_{ss}$$

20 $${\epsilon }_{l}=\frac{{\rm{d}}{\theta }_{l}}{{\rm{d}}t}$$

However, there are two state variable (n = 2) without BTAH: the coverage rate of CuCl and Cu_2_O, which presented as *θ*_1_ and *θ*_2_ respectively. Then the Cu should be 1-*θ*_1_*-θ*_2_.

Then, faradic current density of the whole reaction is:

21 $${I}_{F}={I}_{a}+{I}_{c}={I}_{1}-{I}_{-1}-{I}_{2}={k}_{1}(1-{\theta }_{1}-{\theta }_{2}){\alpha }_{C{l}^{-}}-{k}_{-1}{\theta }_{1}-{k}_{2}{\theta }_{2}{\alpha }_{{O}_{2}}^{1/4}$$

in equation (21), α stand for the corresponding ionic activity.

The value of *θ*_1_ should be increased by the positive reaction of equation (1), but equation (1) reverse reaction and equation (3) should decrease it; *θ*_2_ will be increased by equation (3).

At steady state,

22 $${(\dot{\theta })}_{ss}={k}_{1}(1-{\theta }_{1}-{\theta }_{2}){\alpha }_{C{l}^{-}}-{k}_{-1}{\theta }_{1}-{k}_{3}{\theta }_{1}{\alpha }_{O{H}^{-}}=0$$

*k*_1_ is rate determine step without BTAH^58^, the relation between *θ*_1_, *θ*_2_ and *t* should be expressed by:

23 $${\epsilon }_{1}=\frac{{\rm{d}}{\theta }_{1}}{{\rm{d}}t}=K({k}_{1}(1-{\theta }_{1}-{\theta }_{2}){\alpha }_{C{l}^{-}}-{k}_{-1}{\theta }_{1}-{k}_{3}{\theta }_{1}{\alpha }_{O{H}^{-}})$$

24 $${\epsilon }_{2}=\frac{{\rm{d}}{\theta }_{2}}{{\rm{d}}t}=K({k}_{1}(1-{\theta }_{1}-{\theta }_{2}){\alpha }_{C{l}^{-}})$$

in which *K* is relative coefficient, ϵ_2_ related to the corrosion rate, which limited by *k*_1_.

25 $${m}_{1,1}={(\frac{\partial {I}_{F1}}{\partial {\theta }_{1}})}_{ss}=-{k}_{1}{\alpha }_{C{l}^{-}}-{k}_{-1} < 0$$

26 $${m}_{1,2}={(\frac{\partial {I}_{F1}}{\partial {\theta }_{2}})}_{ss}=-{k}_{1}{\alpha }_{C{l}^{-}} < 0$$

27 $${m}_{2,1}={(\frac{\partial {I}_{F2}}{\partial {\theta }_{1}})}_{ss}=0$$

28 $${m}_{2,2}={(\frac{\partial {I}_{F2}}{\partial {\theta }_{2}})}_{ss}=-{k}_{2}{\theta }_{2}{\alpha }_{{O}_{2}}^{1/4} < 0$$

According to equation (10), *k*_1_ and *k*_−1_ are dependent on *E*, but *k*_3_ is independent to *E*, combined with equations (19) and (23):

29 $${b}_{1}={(\frac{\partial {\in }_{1}}{\partial E})}_{ss}=\frac{KF}{2RT}[{k}_{1}(1-{\theta }_{1}-{\theta }_{2}){\alpha }_{C{l}^{-}}-{k}_{-1}{\theta }_{1}] > 0$$

30 $${b}_{2}={(\frac{\partial {\in }_{2}}{\partial E})}_{ss}=\frac{KF}{2RT}[{k}_{1}(1-{\theta }_{1}-{\theta }_{2}){\alpha }_{C{l}^{-}}] > 0$$

31 $${B}_{1}={m}_{1,1}\cdot {b}_{1}+{m}_{1,2}\cdot {b}_{1} < 0$$

32 $${B}_{2}={m}_{2,1}\cdot {b}_{2}+{m}_{2,2}\cdot {b}_{2} < 0$$

while,

33 $${Y}_{F}=\frac{1}{{R}_{f}}-\frac{|{B}_{1}|}{{a}_{1}+j\omega }-\frac{|{B}_{2}|}{{a}_{2}+j\omega }$$

In this case, the equivalent circuit of the corrosion process is Rs(CPEl(Rf(Rct1CPE2) (Rct2CPE3))), which indicate three capacitive arcs will be displayed on the impedance plane.

After pretreated with BTAH, [Cu(I)BTA]_n_ film takes a function of surface, supposed as *θ*_3_, the covering density of intermediate CuCl and Cu_2_O will be *θ*_1_′ and *θ*_2_′ respectively (*θ*_3_ $\gg$ *θ*_1_′). After ultrasonic cleaning process, *θ*_3_ is a constant and independent to Δ*t* or Δ*E*. In consideration of excellent corrosion protection, equation (3) is restrained, *k*_3_ → 0. $\epsilon {^{\prime} }_{2}={\rm{d}}\theta {^{\prime} }_{2}/{\rm{d}}t=0$, Then Faraday anodic reaction has two time constants: *E* and *θ*_1_′, and cathodic reaction has only one time constants: *E*. the Faraday admittance in equation (15) will be:

34 $${Y}_{F}=\frac{1}{{R}_{f}}+\frac{B^{\prime} }{a^{\prime} +j\omega T}$$

in which,

35 $$b^{\prime} ={(\frac{\partial {\in ^{\prime} }_{1}}{\partial E})}_{ss}=\frac{KF}{2RT}[{k}_{1}(1-{\theta }_{1}-{\theta }_{2}){\alpha }_{C{l}^{-}}-{k}_{-1}{\theta }_{1}] > 0$$

36 $$m^{\prime} ={(\frac{\partial {I}_{F}}{\partial \theta {^{\prime} }_{1}})}_{ss}=-{K}_{1}(1-{\theta }_{3}){\alpha }_{C{l}^{-}}-{K}_{-1}{\theta }_{3} < 0$$

37 $$B^{\prime} =m^{\prime} \cdot b^{\prime}  < 0$$

Then, Y_F_ will be transferred into:

38 $${Y}_{F}=\frac{1}{{R}_{f}}-\frac{|{B}_{1}|}{a+j\omega }=\frac{a+j\omega -{R}_{f}|{B}_{1}|}{{R}_{f}(a+j\omega )}$$

39 $${Z}_{F}=\frac{1}{{Y}_{F}}={R}_{f}+\frac{\frac{{R}_{f}^{2}|{B}_{1}|}{a-{R}_{f}|{B}_{1}|}}{1+j\omega \frac{1}{a-{R}_{f}|{B}_{1}|}}$$

in this way, we suggest:

40 $${R}_{a}=\frac{{R}_{f}^{2}|{B}_{1}|}{a-{R}_{f}|{B}_{1}|}$$

41 $${C}_{a}=\frac{1}{{R}_{f}^{2}|{B}_{1}|}$$

then:

42 $${Z}_{F}=\frac{1}{{Y}_{F}}={R}_{f}+\frac{{R}_{a}}{1+j\omega {R}_{a}{C}_{a}}$$

In this case, the equivalent circuit of the corrosion process is Rs(CPEl(Rf(RctCPE2))), which indicate two capacitive arcs will be displayed on the impedance plane.

Some typical fitting parameters obtained from equivalent circuits of Cu and CuBTA are listed in Tables 2 and 3. The presence of CPE has been explained by dispersion effects that caused by microscopic roughness of the substrate surface, and *n* is the frequency independent parameters of CPE. The decrease of CPE indicate the replacement of water on copper surface by [Cu(I)BTA]_n_ film. On CuBTA, the *n*_1_ parameter (in Table 3) remains approximate quantitative value (0.80~0.82) at each thickness of TEL, which suggests the decreasing dispersion effects, and each corresponding morphology feature is supposed to be smooth and uniform.

**Table 2:** Fitting results of EIS for Cu at different thickness of TEL.

Thickness/μm	1000	400	200	100
*R*_s_/Ω·cm^2^	47.5	31.6	66.4	104.3
*CPE*_1_/F·cm^2^	5.69E-05	6.85E-05	7.47E-05	10.0E-05
n_1_	0.692	0.720	0.719	0.745
*R*_f_/Ω·cm^2^	77.2	73.6	51.4	152
*CPE*_2_/F·cm^2^	7.6E-04	8.8E-04	8.2E-04	7.0E-04
n_2_	0.512	0.482	0.466	0.545
*R*_ct1_/Ω·cm^2^	1428	1734	1643	1195
*CPE*_3_/F·cm^2^	4.25E-03	5.79E-03	6.41E-03	1.43E-03
n_3_	0.702	0.678	0.702	0.859
*R*_ct2_/Ω·cm^2^	2763	2322	1874	1453

**Table 3:** Fitting results of EIS for CuBTA at different thickness of TEL.

Thickness/μm	1000	400	200	100
*R*_s_/Ω·cm^2^	32.5	42.7	59.3	67.8
*CPE*_1_/F·cm^2^	4.09E-06	3.88E-06	3.24E-06	4E-06
n_1_	0.819	0.817	0.810	0.810
*R*_f_/Ω·cm^2^	1416	1312	1220	1235
*CPE*_2_/F·cm^2^	5.35E-05	5.67E-05	6.38E-05	6.05E-05
n_2_	0.653	0.649	0.643	0.665
*R*_ct_/Ω·cm^2^	6370	6183	5980	5720

In the current study, *R*_ct_, which represent corrosion resistance, could also use to estimate the corrosion rate by its inverse proportion relation. The values listed in Tables 2 and 3 verify that copper corrosion rate during its initial stage arranged in the sequence of 100 µm > 200 µm > 400 µm > 1000 µm, which shows good agreement with Tafel results in Table 1.

In some lectures^59,60^, the formation of copper oxide film under the [Cu(I)BTA]_n_ film has been suggested in chloride-containing electrolyte, and in CuBTA, the Cu_2_O layer shows a low p-type conductivity, i.e. it becomes almost an intrinsic semiconductor, that could explain the enlargement of charge transfer resistance (*R*_ct_) in Table 3.

### Electrochemical noise analysis

**Figure 8:**
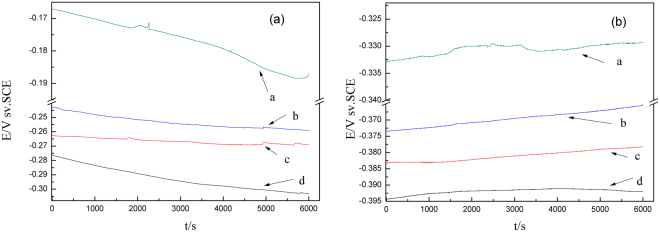
Electrochemical noise data of (**a**) Cu and (**b**) CuBTA under different thickness of TELs of 3.5 wt.% NaCl at 20 °C: a – 100 μm; b – 200 μm; c – 400 μm; d – 1000 μm. The sampling interval was 0.25 s.

The EN technique has carried out to test Cu and CuBTA, for the purpose to qualitatively analyze the relationship between the EN features and the corrosion severity, and the results are shown in Fig. 8. The potential curves recorded in Fig. 8 are characterized by smoothness, which is generated by the steady diffusion-controlled process^61^.

**Figure 9:**
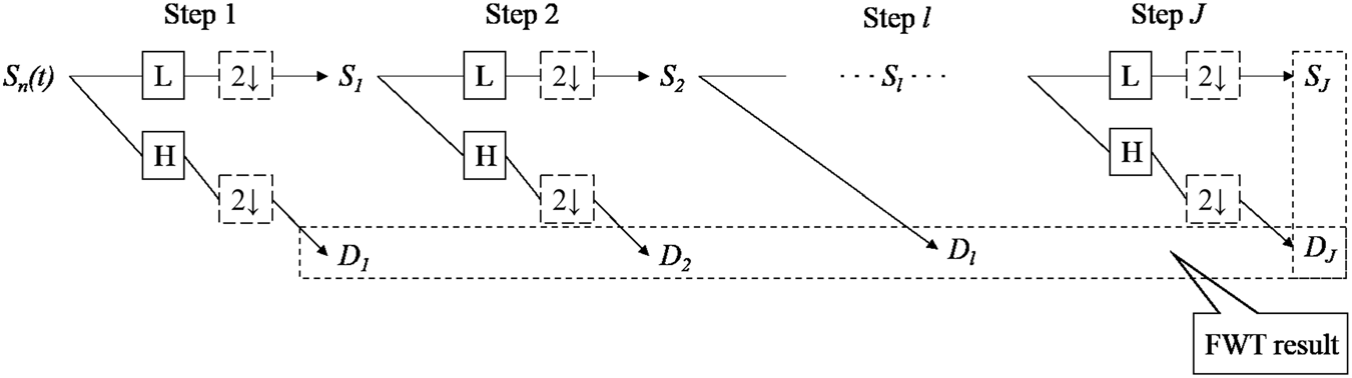
Theoretical algorithm scheme of the fast wavelet transform. J = 8 is often chosen for electrochemical noise experiment.

Fast Wavelet Transformation (FWT) technique of the fourth order is used to achieve the energy distribution plot (EDP), which theoretical algorithm is depicted in details in Fig. 9. Briefly, the real time signal sets *S*_n_(t) (n = 1,2,…*N*) is decomposed into two sets of coefficients: a smooth coefficient set, *S* = (*S*_1_, *S*_2_, …, *S*_J_), which contains the information about the general trend of the signal; a detail coefficient set, *D* = (*D*_1_, *D*_2_, …, *D*_J_), which contain the information about the local fluctuations in the signal^62^. In FWT analyzes, *D*_1_, *D*_2_…*D*_J_ and *S*_J_ are designated as the so-called “crystals”.

Based on electrochemical noise energy (*E*_N_) calculated, the general relations could be obtained:

43 $${E}_{j}^{D}=\sum _{k=1}^{N/{2}^{j}}{D}_{j,k}^{2},(j=1,2,\ldots ,J)$$

44 $${E}_{J}^{S}=\sum _{k=1}^{N/{2}^{j}}{S}_{J,k}^{2},(j=1,2,\ldots ,J)$$

45 $${E}_{N}=\sum _{j=1}^{J}{E}_{j}^{D}+{E}_{J}^{S}$$

**Figure 10:**
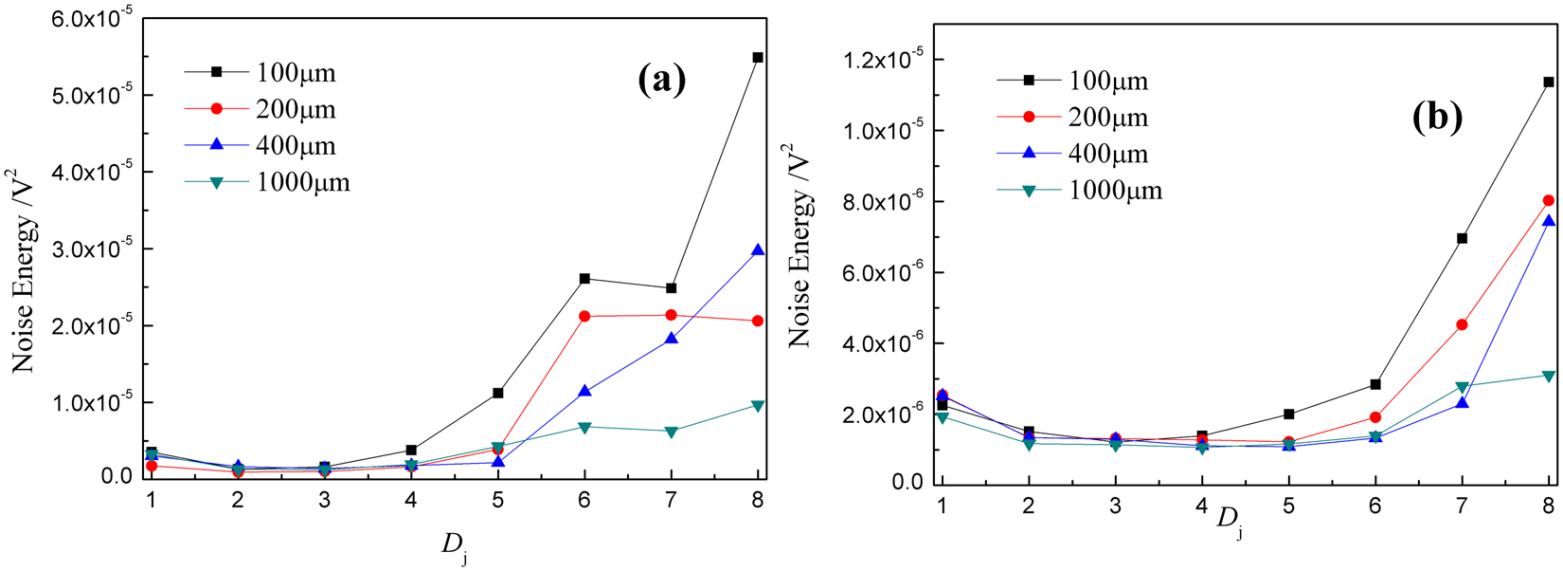
RP-EDP map at different thickness of 3.5 wt.% NaCl thin electrolyte film: (**a**) Cu and (**b**) CuBTA.

As previous report^36,41,63^, *J* = 8 is often chosen for study, and EDP map was often replotted by discounting the contribution of *S*_8_ coefficients to the overall ensemble signal energy. The replotted EDP (RP-EDP) map can be divided into three segments, thus, the distinct type of events of electrochemical noise can be distinguished by their different time constant: (1) region I between *D*_1_ and *D*_3_ in the higher frequency mainly characterizes a reasonably fast phenomenon, such as metastable pitting and nucleation process, (2) region II between *D*_4_ and *D*_6_ mainly characterizes the growth process, and (3) region III between *D*_7_ and *D*_8_ at lower frequency mainly reflects the information about the diffusion process, and it has also been verified by other literatures^64,65^. In this paper, the RP-EDP maps without normalization at different thickness of TELs are replotted in Fig. 10, which indicate significantly large low-frequency contribution, i.e. presence of large timescale processes.

Therefore, the energy sum of *D*_1_ − *D*_6_ (*E*_*c*_, in the unit of V^2^), which is defined as the active energy of corrosion reactions, should reflect the metastable pitting nucleation and growth energy, i.e. the corrosion severity.

46 $${E}_{c}={E}_{1}^{D}+{E}_{2}^{D}+{E}_{3}^{D}+{E}_{4}^{D}+{E}_{5}^{D}+{E}_{6}^{D}$$

It should be noticed that, the general trend of the signal takes most fraction energy of tested noise. The *E*_c_ represent the energy sum of flicker noise caused by nucleation and growth of corrosion pits at a particular testing frequency^66^.

**Figure 11:**
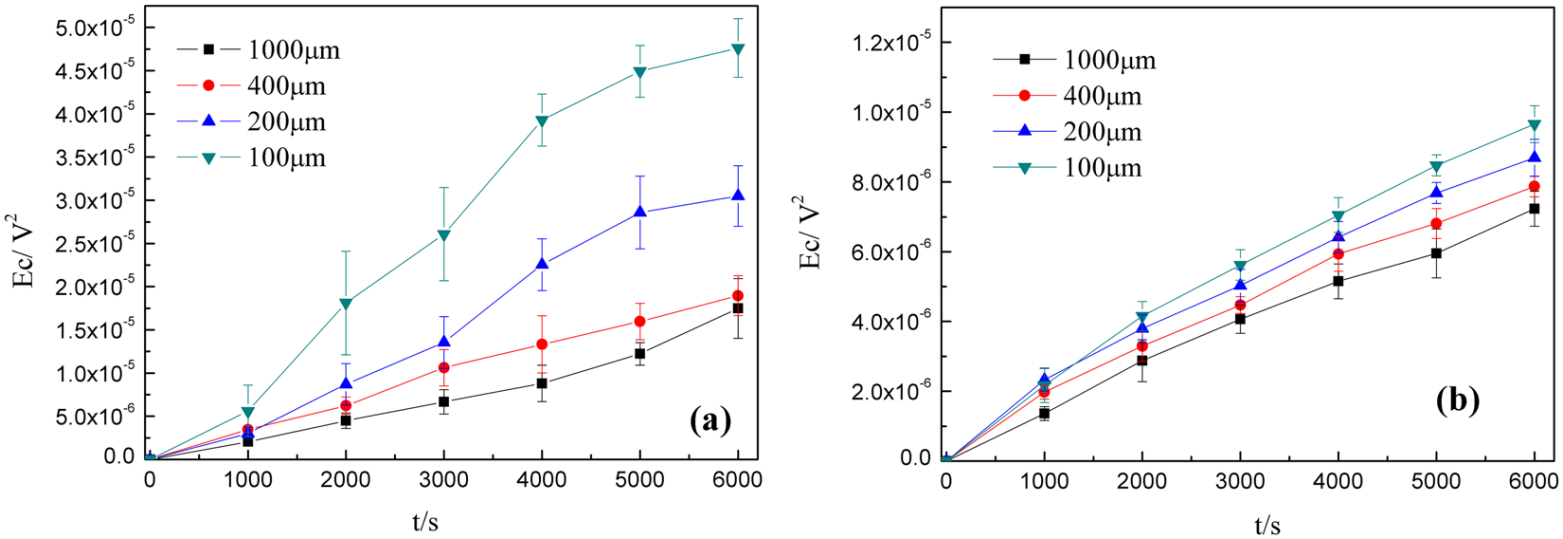
The relationship between corrosion energy (*E*_c_) and time at different thickness of thin electrolyte film: (**a**) Cu and (**b**) CuBTA.

The tested corrosion energy variation with time under different thickness of electrolyte has listed in Fig. 11. The slope of each line in Fig. 11 is defined as energy density (d*E*_c_/dt) during corrosion process, and the average values are listed in Table 4.

**Table 4:** The average *E*_c_ density value (d*E*_c_/dt) at different thickness of TEL.

Thickness/μm	Average *E*_c_ density value (d*E*_c_/dt)/V^2^·s^−1^			
	100	200	400	1000
Cu	8.67E-09	5.59E-09	3.18E-09	2.75E-09
CuBTA	1.78E-09	1.41E-09	1.28E-09	1.25E-09

**Figure 12:**
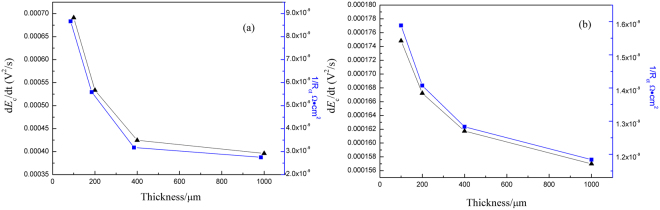
Relationship between 1*/R*_ct_ and average corrosion energy density (d*E*_c_/d*t*) at different thickness of 3.5 wt.% NaCl TELs: (**a**) Cu and (**b**) CuBTA.

On the purpose to qualitatively analyze the EN energy, Fig. 12 denotes the relationship between 1*/R*_ct_ (obtained by EIS) and the average corrosion energy density value (d*E*_c_/d*t*) at each TEL with and without pretreatment of BTAH. Apparently, the d*E*_c_/d*t* shows the direct variation trend with 1*/R*_ct_. which denotes the corrosion rate.

This result also reveals that RP-EDP maps without normalization can not only used as fingerprint to characteristic the morphology, but also speculate the corrosion energy *E*_c_ to deduce corrosion rate properly. Comparing to the traditional parameters of corrosion rate, such as weight loss or corrosion current density, the electrochemical noise offers a nondestructive on-line monitoring progress which can be easily carried out, besides, the speculated parameter *E*_c_ demonstrates closer link to the surface microstructure and represents the corrosion rate and severity.

### Surface analysis

**Figure 13:**
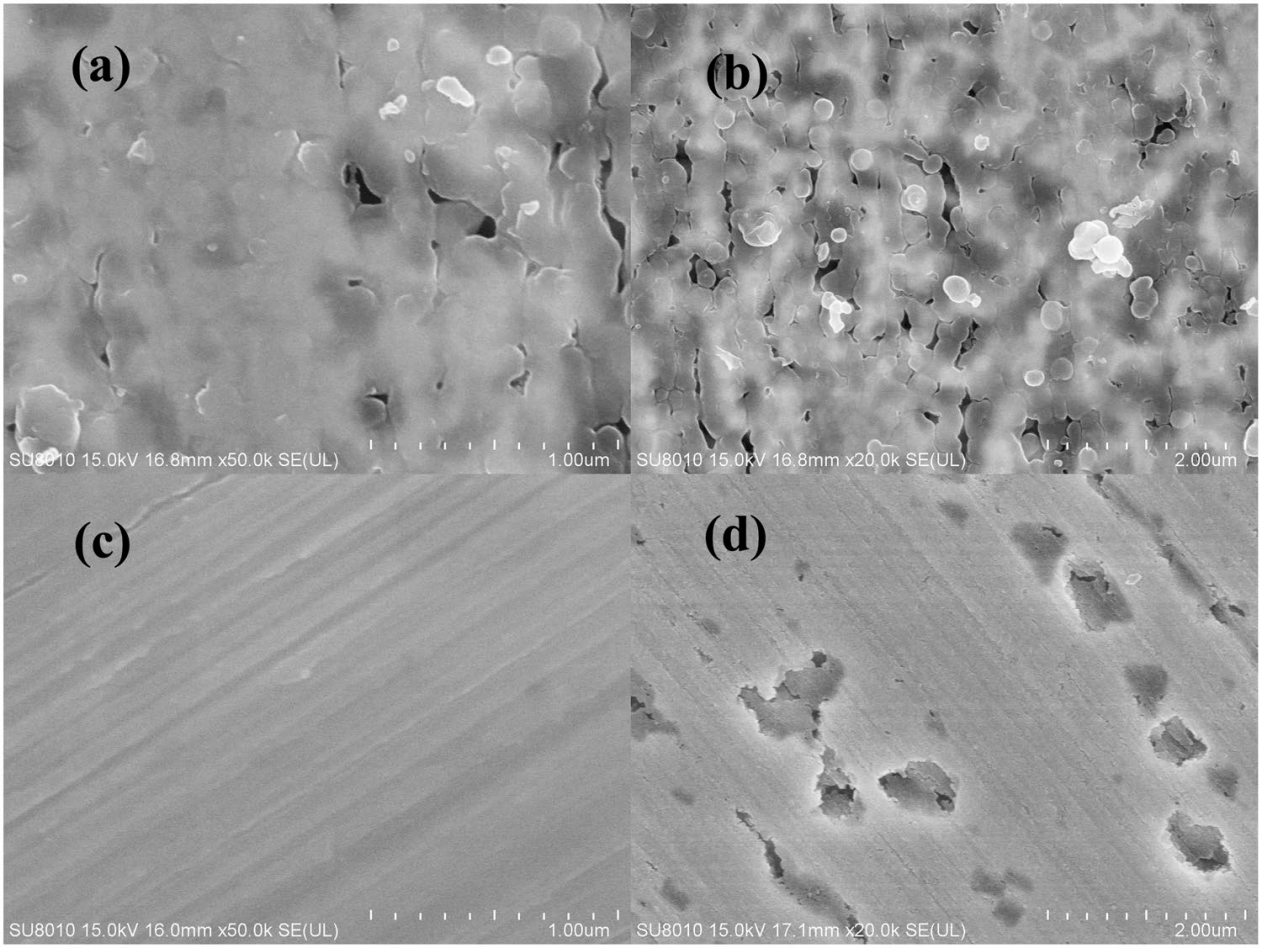
SEM image of samples: (**a**) CuBTA without corrosion, (**b**) CuBTA after 100 min’s corrosion under 100 μm 3.5 wt.% NaCl TEL, (**c**) Cu without corrosion and (**d**) Cu after 100 min’s corrosion under 100 μm 3.5 wt.% NaCl TEL.

Figure 13 shows the micrographs of Cu and CuBTA, and the corresponding EDS analysis results are listed in Table 5. CuBTA shows a covering layer deposits surface in Fig. 13a, which definitely related to be [Cu(I)BTA]_n_ film by raised C and N element relative weight in Table 5. The morphology of [Cu(I)BTA]_n_ film seems to be smooth and it’s almost entirely covering the copper surface except a few black cracks left. After 100 min’s corrosion, the Cl^−^ ion pass through the cracks on the film and attack the Cu substrate and a few cracks are amplified (Fig. 13b), whereas the corrosion process are inhibited by the inherent film.

**Table 5:** Element content deduced from EDS of copper under 3.5 wt.% NaCl TEL.

	C (wt.%)	N (wt.%)	O (wt.%)	Cl (wt.%)	Cu (wt.%)
CuBTA	4.514	1.065	0.893	0.033	93.495
CuBTA after corrosion	4.273	1.024	1.063	0.056	93.483
Cu	0.049	0.076	1.158	0.076	98.841
Cu after corrosion	0.000	0.047	1.814	0.070	98.069

Cu surface without corrosion is smooth and uniform in Fig. 13c, whereas, the occurred corrosion pits after corrosion (Fig. 13d) denote intensive corrosion. Besides, the Cl element remain the same value after corrosion, hence, the main initial corrosion products of copper in chloride-containing TEL supposed to be cuprous oxide, which is accordance to other reports^11,12,67^.

## Conclusion

The initial corrosion process of copper and the corrosion resistance mechanism of Benzotriazole (BTAH) under chloride-containing thin electrolyte layer (3.5 wt.% NaCl) was investigated using Tafel curves, EIS, XPS and electrochemical noise measurement. The results showed that, BTAH was chemisorption onto copper surface through Cu-N bond tightly, leading to [Cu(I)BTA]_n_ film which had verified by theoretical calculation and experimental characterization. Corrosion rate (1*/R*_ct_) increased as thin electrolyte layer thickness decreases which controlled by diffusion-controlled process.

The corrosion energy (*E*_c_) deduced from electrochemical noise served as another calculation for corrosion severity, the *E*_c_ increased as the decreasing thin electrolyte layer thickness, and existed direct proportion to the corrosion rate 1/*R*_ct_. The correlation between *E*_c_ and corrosion rate denoted feasibility to determine corrosion rate by nondestructive on-line monitoring electrochemical noise progress.

## Electronic supplementary material

Supplementary Information
