# Supplementary material for: Adsorption and protective behavior of BTAH on the initial atmospheric corrosion process of copper under thin film of chloride solutions
Source: Sci Rep. 2018 Apr 4;8:5606. doi: 10.1038/s41598-018-23927-w (PMC5884792; doi:10.1038/s41598-018-23927-w)
Supplement: Supplementary file 1 — Supplementary Information [file 41598_2018_23927_MOESM1_ESM.pdf]

Supplementary Information

Adsorption and protective behavior of BTAH on the initial atmospheric corrosion process of copper under thin film of chloride solutions

Chenxi Yi[a], Benfeng Zhu[a], Yu Chen[b], Xiaoqing Du[a], Yumeng Yang[a], Jiao Liu[c], Zhao Zhang[a,*]

[a] *Department of Chemistry, Zhejiang University, Hangzhou, Zhejiang 310027, China*

[b] *Department of Chemical Engineering and Safety, Binzhou University, Binzhou, Shandong 256600, China*

[c] *College of Chemistry and Chemical Engineering, Hunan University, Changsha 410082, China*

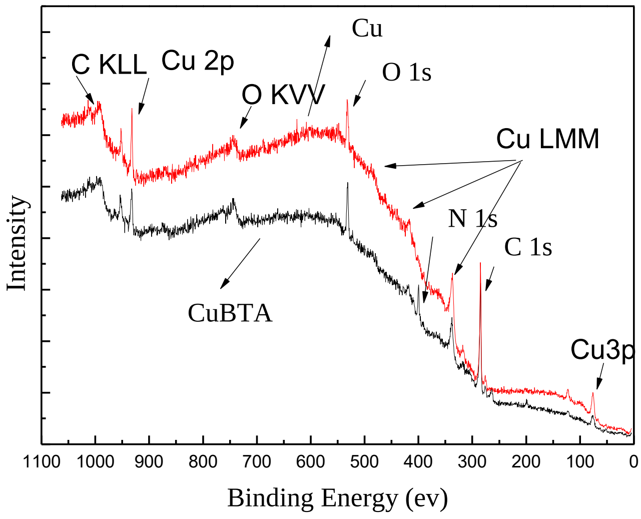

Figure S1 The XPS spectra of CuBTA and Cu electrode after 100 min's corrosion at a range of 0 ~ 1100 eV. The electrode emerged in 3.5 wt.% NaCl at 60 ºC.

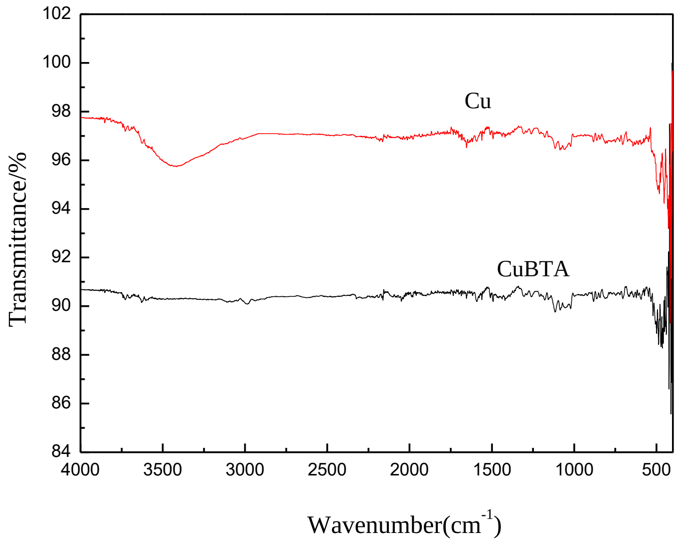

Figure S2 FTIR spectra of CuBTA and Cu after 100 min's corrosion under 100 μm TEL of 3.5 wt.% NaCl. Ranged from 400 ~ 4000 cm^{-1}, and 32 scans were performed for each spectrum with a resolution of 2 cm^{-1}.
